# Strategies for increasing diagnostic yield of community-onset bacteraemia within the emergency department: A retrospective study

**DOI:** 10.1371/journal.pone.0222545

**Published:** 2019-09-12

**Authors:** Kathrin Rothe, Christoph D. Spinner, Armin Ott, Christiane Querbach, Michael Dommasch, Cassandra Aldrich, Friedemann Gebhardt, Jochen Schneider, Roland M. Schmid, Dirk H. Busch, Juri Katchanov

**Affiliations:** 1 Technical University of Munich, School of Medicine, Institute for Medical Microbiology, Immunology and Hygiene, Munich, Germany; 2 Technical University of Munich, School of Medicine, University Hospital rechts der Isar, Department of Medicine II, Munich, Germany; 3 Technical University of Munich, Institute of Medical Informatics, Statistics, and Epidemiology, Munich, Germany; 4 Technical University of Munich, School of Medicine, University Hospital rechts der Isar, Pharmacy Department, Munich, Germany; 5 Technical University of Munich, School of Medicine, University Hospital rechts der Isar, Department of Medicine I, Munich, Germany; 6 Ludwigs-Maximilians-University Munich, Division of Infectious Diseases and Tropical Medicine, Munich, Germany; 7 German Centre for Infection Research (DZIF), Partner Site Munich, Munich, Germany; University of Mississippi Medical Center, UNITED STATES

## Abstract

Bloodstream infections (BSI) are associated with high mortality. Therefore, reliable methods of detection are of paramount importance. Efficient strategies to improve diagnostic yield of bacteraemia within the emergency department (ED) are needed. We conducted a retrospective analysis of all ED encounters in a high-volume, city-centre university hospital within Germany during a five-year study period from October 2013 to September 2018. A time-series analysis was conducted for all ED encounters in which blood cultures (BCs) were collected. BC detection rates and diagnostic yield of community-onset bacteraemia were compared during the study period (which included 45 months prior to the start of a new diagnostic Antibiotic Stewardship (ABS) bundle and 15 months following its implementation). BCs were obtained from 5,191 out of 66,879 ED admissions (7.8%). Bacteraemia was detected in 1,013 encounters (19.5% of encounters where BCs were obtained). The overall yield of true bacteraemia (defined as yielding clinically relevant pathogens) was 14.4%. The new ABS-related diagnostic protocol resulted in an increased number of hospitalised patients with BCs collected in the ED (18% compared to 12.3%) and a significant increase in patients with two or more BC sets taken (59% compared to 25.4%), which resulted in an improved detection rate of true bacteraemia (2.5% versus 1.8% of hospital admissions) without any decrease in diagnostic yield. This simultaneous increase in BC rates without degradation of yield was a valuable finding that indicated success of this strategy. Thus, implementation of the new diagnostic ABS bundle within the ED, which included the presence of a skilled infectious disease (ID) team focused on obtaining BCs, appeared to be a valuable tool for the accurate and timely detection of community-onset bacteraemia.

## Introduction

Bacteraemia is a public health concern with high attributable morbidity and mortality with estimated annual deaths exceeding 150,000 in Europe and 80,000 in North America [1, 2]. The majority of patients with bacteraemia are admitted to the hospital through the emergency department (ED) [3]. However, given the low diagnostic yield, the clinical utility of obtaining blood cultures (BCs) in the ED has been questioned in recent literature [4, 5]. Nevertheless, BCs remain the gold standard and the most important first-line tool for diagnosing severe bacterial infections [6–9]. Optimising the yield of true bacteraemia from blood cultures is crucial, as negative blood cultures are costly and detection of contaminants results in overtreatment [10]. Several rules for prediction of true positive bacteraemia, which is defined as yielding relevant pathogens and not merely contaminants, have been proposed, but none are universally accepted [3, 11–14]. Remarkably, elevated body temperature (fever) is an unreliable clinical indicator of true positive bacteraemia [15, 16].

Collected blood volume and the likelihood of detecting bacteraemia are linked. Therefore, current guidelines recommend that 2–4 blood culture sets (BCS) should be obtained, including paired aerobic and anaerobic bottles, and that one set should contain 8–10 mL of whole blood per bottle [8, 17–19]. It is widely accepted, that the majority of BCs are critically under-filled, containing less than 5 mL of blood [8, 20, 21] and that BCs are also at risk of high contamination rates, particularly in emergency settings [22, 23]. The identification of a relevant microorganism in the bloodstream facilitates the search for an infectious focus and allows adjustment to a directed, narrower-spectrum antimicrobial therapy. Therefore, accurately obtained BCs provide a diagnostic basis for implementing antibiotic stewardship (ABS) in the ED [10, 24].

Diagnostic stewardship is an integral part and the basis for ABS intervention, as appropriate use of microbial diagnostics and a close partnership between clinicians and laboratorians results in a more sophisticated diagnosis and therefore can optimise patient management [25–27]. Multidisciplinary approaches for diagnostic stewardship as part of an ABS program include the development of local guidelines for specimen collection, regular training, team meetings, and effective communication with the microbiologist, including on-call services for follow-up requests [25].

In the ED setting, severe bloodstream infections (BSI) must be reliably detected in order to correctly identify patients requiring further diagnostics or therapeutic interventions. This study investigated the effect of a diagnostic ABS intervention strategy in improving the use of BCs within the ED. We analysed data from two time periods, prior to and after the implementation of the diagnostic ABS bundle in the ED of a large city-centre university hospital in Munich, Germany.

## Materials and methods

A descriptive, retrospective analysis of all BCs collected in the internal medicine and neurology ED of a city-centre university hospital with approximately 1,200 beds in Munich, Germany between the 1^st^ October 2013 and 31^st^ September 2018 was performed. The ED provides primary care to patients with all non-surgical emergencies, resulting in approximately 14,000 patient visits per year. A stable 45-month time period prior to the implementation of the diagnostic ABS bundle (pre-diagnostic ABS period) was compared to a 15-month period following implementation of the diagnostic ABS bundle (diagnostic ABS period).

### Inclusion and exclusion criteria

All ED encounters with BCs taken were included in our retrospective study. The inclusion criteria were as follows: (1) age at the time of ED encounter > 18, and (2) BC samples collected and processed in the Department of Microbiology. BC samples that were discarded due to the establishment of an alternative non-infectious disease (ID) diagnosis were excluded.

### Collecting BC samples

Blood was obtained at bedside exclusively by physicians (mainly internal medicine residents), according to national practice after local skin decontamination using octenidin-hydrochloride-based skin disinfection. Whole blood samples (8–10 mL per bottle) were inoculated into aerobic and anaerobic blood culture media (BACTEC™ Plus aerobic and anaerobic medium, Becton Dickinson, Sparks, MD, USA), suitable for processing via an automated blood culture system (BACTEC™ Fluorescent Series, Becton Dickinson). Bottles were incubated for 5 to 7 days according to the manufacturer’s recommendations. Immediate Gram stain identification, species identification (MALDI-TOF MS, Bruker Daltronics GmbH, Leipzig, Germany), and automated antimicrobial susceptibility testing (VITEK®, bioMerieux, Marcy l’Etoile, France) were performed for all positive cultures.

In ED encounters with more than one BCS taken, the sets were not numbered (e.g. “first BCS”, “second BCS”). As diagnostic yield is highly dependent on the overall blood volume drawn [4], the number of sets was used as a surrogate for the volume taken.

All BC samples were drawn during the patient’s stay in the ED. Physicians were encouraged to take BCs as soon as possible and in the majority of cases BCs were taken near the time of the initial blood investigations. There was no formal recommendation regarding the procedure and no retrospective analysis of the timing.

### Definition of true bacteraemia

All of the isolates were categorised as yielding relevant pathogens (= true positives) or contaminants by dichotomous decision-making following assessment by at least two investigators, including a clinical microbiologist and a clinical physician. In order to discern whether recovered pathogens represented contaminants (such as common skin commensals like coagulase-negative staphylococci) from a single culture, isolates were reviewed separately on the basis of the number of positive BCS. The patients’ characteristics and the presence of intravascular devices or indwelling catheters were also considered. If at least two bottles out of two or three BCS yielded the same coagulase-negative staphylococci, the isolates were considered clinically significant. Disagreements were to be adjudicated by a third clinical physician; however, no disagreements occurred. BCs that grew contaminants only were defined as false positives (contamination).

The detection rate of bacteraemia and the diagnostic yield of BCs were calculated according to the definitions in Table 1. To determine the detection rates of bacteraemia and diagnostic yield, the number of ED encounters was used as the denominator throughout the study.

**Table 1:** Definitions.

Detection rate of true bacteraemia	Number of hospitalised ED encounters with true-positive BCs / number of all hospitalised ED encounters
Detection rate of false bacteraemia (contamination)	Number of hospitalised ED encounters with BCs that exclusively grew contaminants / number of all hospitalised ED encounters
Diagnostic yield of BC	Number of hospitalised ED encounters with true-positive BCs / number of all hospitalised ED encounters with BCs obtained

### Diagnostic ABS intervention

There were no pre-existing, documented clinical guidelines for BC sampling at the hospital available. From July 2017 to September 2018 (diagnostic ABS intervention period), a diagnostic ABS bundle was implemented with the following elements focused on improving the use of BCs in the ED:

1. Rotation of an ID resident to work a full shift within the ED. The ID resident worked 12-hour day and night shifts along with the residents from other departments. Although there was no formal educational role, the ID resident supervised the implementation of the ABS bundle, advised fellow residents in all issues concerning ID, and established an additional personal link to the diagnostic Department of Microbiology. ED physicians were explicitly encouraged by the ID resident to collect two sets of BCs and to generally obtain BCs very liberally. In cases where two or more BCS were obtained, the actual sampling strategy, such as multiple venepunctures or collecting multiple BCS through one single puncture was at the discretion of the physician performing the procedure. We accepted “single-sampling strategies”, i.e. collecting the total volume of blood from one single draw, as one of the possible strategies for BC collection in the ED. This strategy satisfies the need to collect a sufficient volume of blood and the need to decrease contamination rates by limiting the number of punctures. In a busy ED environment, this strategy is also associated with reduced workload, improved comfort for patients, reduced risk of omitting subsequent draws, and early initiation of empirical antibiotic treatment [8, 28–31].
2. Development of local standard operation procedures (SOP) for obtaining BCs and regular visits by an ABS team, including a clinical microbiologist and a clinical pharmacist, to implement the local guidelines. Within the provided SOP, physicians were advised to obtain BCs if one or more of the following major indications were present:
  (A) Clinically suspected organ infection with accompanying bacteraemia, such as meningitis, cholecystitis, pyelonephritis, necrotising fasciitis, osteomyelitis, severe pneumonia, endocarditis, vascular graft infection, or prosthetic joint infection
    OR
  (B) Suspected sepsis, defined by the sepsis-related organ failure assessment score, quickSOFA (qSOFA), consisting of a respiratory rate of 22/min or greater, altered mentation, or systolic blood pressure of 100 mmHg or less [32]
    OR
  (C) Detected or reported body core temperature ≥ 38.3°C.
    As elevated body temperature is an unreliable clinical indicator of bacteraemia, awareness was raised to obtain BCs in the absence of an elevated temperature. Physicians were also taught to obtain BCs very liberally in every clinically suspected systemic infection, not exclusively focussing on these major indications. If the suspected infection was not clinically confirmed or could be dismissed after laboratory results, BCs should then be discharged without further processing.
3. Expedited processing and reporting of positively flagged BCs: subcultures were incubated briefly on solid growth media, and species identification (via MALDI-TOF) was performed within 4–6 hours after a positive signal from a BC bottle [33]. The clinical microbiologist of the ABS team called the ED residents daily and was available during working hours for all enquiries.

### Statistics

Quantitative data are described as mean ± standard deviation, and categorical measures are presented as absolute and relative frequencies. Chi-squared tests were used for comparison of rates between the two strategies. A moving average estimator with n = 4 was used to illustrate the change in the rates over time and to reduce the effect of seasonality. All tests were performed two-sided with a level of significance (alpha, α) of 5%. Statistical analyses were performed using the statistical software R (version 3.5.2; R Foundation for Statistical Computing, Vienna, Austria).

### Ethics

The Ethics Committee of the Technical University of Munich approved the protocol for this retrospective study and waived the need to obtain consent for the collection, analysis, and publication of the retrospectively obtained and anonymised data (approval number 539/18S).

## Results

### ED encounters

During the study period, 66,879 patient encounters within the ED were recorded. Presentations through ambulance and emergency medical services (EMS) accounted for 35% of all ED encounters and self-presenting, walk-in patients accounted for the remaining 65%. A total of 37,850 presentations resulted in admission to the hospital (56.6%). The mean age of all ED encounters was 54.2 years (± 20.8 years). Female and male patients were equally represented in the ED (49.8% and 50.2%, respectively). BCs were obtained at 5,191 ED encounters, of which 1,013 (19.5%) had positive results; contaminants alone were documented in 266 cases (5.1%), and fungaemia was detected in 7 cases (0.1%). In 740 ED cases, true bacteraemia was detected, resulting in a BC diagnostic yield of 14.3% (740/5191).

### Microbiological findings

The microorganisms most frequently isolated from BC samples were gram-negative Enterobacteriales (n = 439), dominated by *Escherichia coli* (n = 376), followed by *Klebsiella pneumoniae* (n = 48). The most common gram-positive microorganism detected in cases of true bacteraemia was *Staphylococcus aureus* (n = 92). Forty-two cases (5.6% of true bacteraemia episodes) were found to be polymicrobial.

Anaerobic organisms represented only a small fraction of true bacteraemia episodes (2.7%, 20 episodes), with *Bacteroides fragilis* (n = 10) being the most frequently isolated anaerobic species. However, in 89 episodes (11.9% of true bacteraemia episodes), the causative pathogen exhibited growth in the anaerobic medium bottle only. Of these episodes, *Escherichia coli* (n = 44) was the most frequently isolated pathogen.

The number of cases of community-onset fungaemia was very low. During the study period, only 7 episodes of community-onset fungemia were detected, among which Candida albicans (n = 3), Candida parapsilosis (n = 2), and Candida tropicalis (n = 2) were identified.

### Impact of the new diagnostic ABS intervention bundle

**Fig 1:**
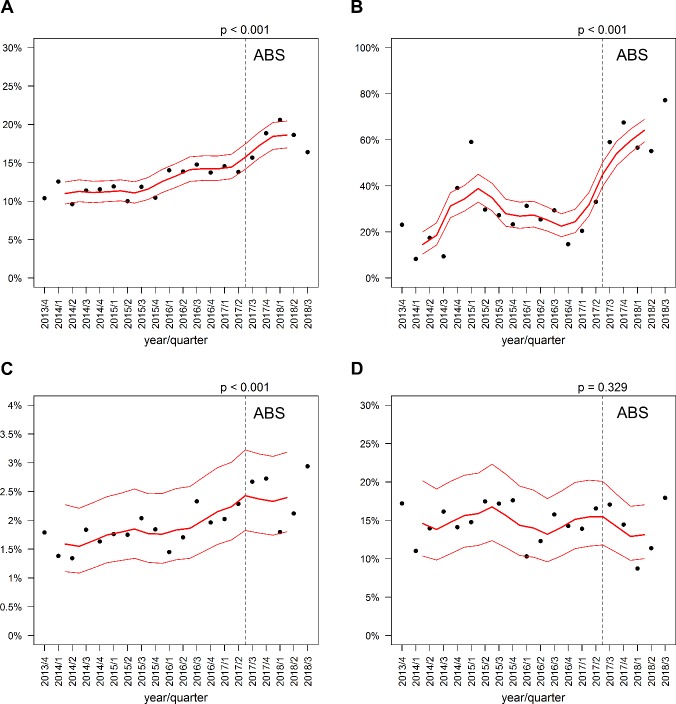
Impact of the diagnostic ABS bundle in the ED. (A) Percentage of hospitalised ED encounters with BCs obtained. (B) Percentage of ED encounters with BCs obtained with two or more BCS collected. (C) Detection rate of bacteraemia in hospitalised ED encounters. (D) Diagnostic yield of true bacteraemia. The dotted line represents the introduction of the new diagnostic ABS bundle. The red line represents the moving average estimator with n = 4 and its 95% confidence interval. *P*-values were obtained by chi-squared tests.

Implementation of the diagnostic BC bundle in the ED resulted in a higher proportion of absolute BC collection, an increase in the number of BCS taken per patient, and an improved detection rate of true bacteraemia without any decrease in diagnostic yield (Table 2 and Fig 1). This simultaneous increase in BC rates without degradation of yield is a valuable finding indicating the success of the strategy.

**Table 2:** BC performance pre- and post-implementation of a diagnostic stewardship strategy in the ED.

	Oct 2013–Jun 2017 (pre-diagnostic ABS intervention period)	Jul 2017–Sep 2018 (diagnostic ABS intervention period)	Chi-squared test for difference in rates between time periods
Hospitalised ED encounters with BCs obtained in the ED/ all hospitalised ED encounters (%)	3,476 / 28,313 (12.3%)	1,715 / 9,537 (18.0%)	*p*<0.001
ED encounters with ≥ 2 BCS obtained/ ED encounters with BCs obtained	920 / 3,476 (26.5%)	1,080 / 1,715 (63.0%)	*p*<0.001
Detection rate of true bacteraemia	513 / 28,313 (1.8%)	234 / 9,537 (2.5%)	*p*<0.001
Diagnostic yield of BC	513 / 3,476 (14.8%)	234 / 1,715 (13.6%)	*p* = 0.329

### Potential effect of the diagnostic ABS intervention bundle on the adjustment of antimicrobial therapy

**Fig 2:**
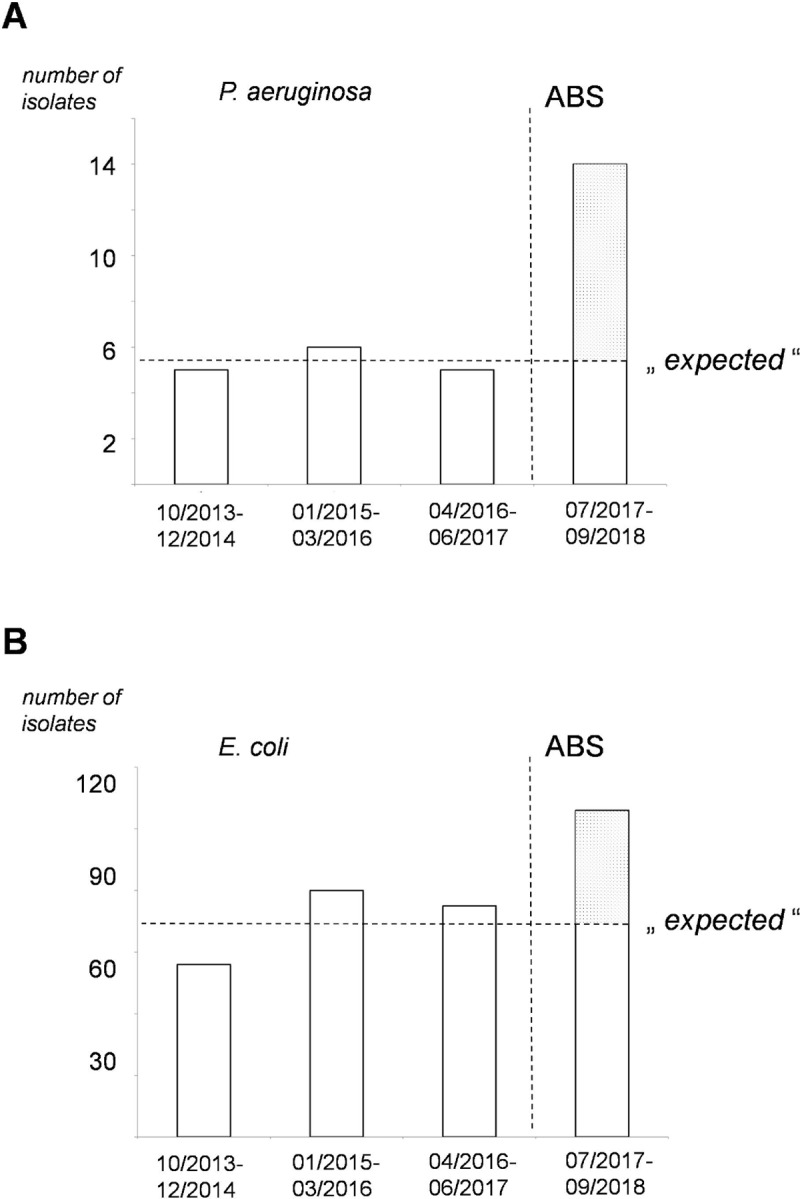
Expected and actually detected episodes of bacteraemia during the diagnostic ABS-period. Number of detected isolates of *P*. *aeruginosa* (A) and *E*. *coli* (B) before and after implementation of the diagnostic ABS bundle (vertical dotted line). Each bar represents a 15-month period. The horizontal dotted line indicates the expected number of isolates in the 15-month diagnostic ABS period based upon the means for the three 15-month periods prior.

We extrapolated the average number of bacteraemia episodes from the period prior to the implementation of the diagnostic ABS bundle in order to calculate the expected number of bacteraemia episodes during the diagnostic ABS period. The expected number was the average of detected episodes in the 15-month time period prior to diagnostic intervention for the particular microorganism. Table 3 displays the numbers of expected episodes of bacteraemia and the actual detected episodes during the diagnostic ABS period for the main causative organisms of true bacteraemia. The actual number of detected bacteraemia cases exceeded the expected number during the diagnostic ABS period for *Pseudomonas aeruginosa* and *Escherichia coli* (Fig 2 and Table 3). Only slight variation in the absolute numbers of detected isolates over the 45-month period prior to the diagnostic intervention was observed, suggesting a stable patient collective. During the diagnostic ABS intervention period, 36 “additional” episodes of *Escherichia coli* bacteraemia were detected (i.e. episodes over the expected extrapolated number). Sixteen *Escherichia coli* isolates were susceptible to ampicillin, and one isolate was susceptible to ampicillin-sulbactam, allowing potential de-escalation of empiric therapy to a narrower-spectrum therapy. There were eight “additionally” detected *Pseudomonas aeruginosa* bacteraemia episodes. All but one isolate were susceptible to piperacillin-tazobactam and 3^rd^ generation cephalosporins (such as ceftazidime), potentially allowing monotherapy with one of these beta-lactam antimicrobials.

**Table 3:** Additionally detected microorganisms during the 15-month diagnostic ABS intervention period.

Microorganism	Expected number of episodes during the diagnostic ABS intervention period (average number of detected episodes in the pre-diagnostic ABS intervention period)	Actual number of detected episodes during the diagnostic ABS intervention period
*Staphylococcus aureus*	23	23
*Streptococcus pneumoniae*	8	10
*Escherichia coli*	70	106
*Klebsiella pneumoniae*	12	12
*Pseudomonas aeruginosa*	6	14

In order to study the effects of obtaining two BCS on the detection of *Escherichia coli* and *Pseudomonas aeruginosa* during the diagnostic ABS period, we examined all episodes positive for these two microorganisms where BCS were taken. Out of the 47 episodes of *Escherichia coli* bacteraemia, in 41 episodes (87.2%), both BCS were positive. Retrospectively, in these episodes, 1 BCS would have been sufficient. However, adding a second set enabled detection in the remaining six episodes (12.8%). Out of the six episodes of *Pseudomonas aeruginosa* bacteraemia with two BCS obtained, in three episodes, *Pseudomonas aeruginosa* growth could be detected in only one of the two sets. Here, a notable proportion of *Pseudomonas aeruginosa* bacteraemia episodes might have been missed if only one BCS had been obtained.

## Discussion

According to previous studies, bacteraemia was detected in 10 out of 1000 hospital admissions in Europe, the U.S., and Taiwan [1, 3, 34–36]. However, the detection rate of bacteraemia in hospitalised ED encounters in our study was higher and was increased from 18 to 25 out of 1000 admissions by the diagnostic ABS intervention bundle. As part of an ABS strategy, diagnostic stewardship interventions focused on the appropriate use of microbial diagnostics and a close partnership between clinicians and laboratorians resulted in more sophisticated diagnosis and optimisation of patient management [25–27].

Our study suggested that diagnostic ABS intervention was capable of increasing the recovery rate for true bacteraemia while simultaneously increasing the overall numbers of BCs obtained and the number of multiple (more than two) BCS collected per patient, which increased the detection rate at stable contamination rates. Further, this finding has important practical implications, as detection of the relevant causal microorganism in bacteraemia facilitates identification of the infectious focus, which can improve targeted antibiotic treatment and enable de-escalation of broad-spectrum empiric antimicrobial therapy. Improving the detection rate of bacteraemia in EDs is particularly important in view of the increasing worldwide incidence of infections caused by multidrug-resistant gram-negative bacteria, resulting in the primary need for empiric regimens covering gram-negative rods using broad-spectrum antimicrobials [37]. Importantly, most of the additionally recovered pathogens in our study were gram-negative pathogens, and their identification allows possible tailoring of an appropriate antimicrobial therapy from empiric broad-spectrum antimicrobial treatment to an agent with the narrowest spectrum. A further study focused on measuring this tailoring effect upon antibiotic therapy in the clinical setting which includes patient outcomes should be conducted in the future.

Optimising the yield of true bacteraemia from BCs is crucial, as negative blood cultures are costly and the detection of contaminants rather than the true pathogen results in overtreatment with an unnecessary risk of side effects for the patient [10].

One of the main criticisms for obtaining BCs in the ED is the low diagnostic yield. Hence, strategies to improve the diagnostic yield and studies aimed at improving BC yield, such as the implementation of educational programs based on proper BC collection, are warranted [38]. It has been reported that physicians overestimate the likelihood of bacteraemia for many of their patients [39–41]. One study showed a diagnostic BC yield in the ED as low as 2.8% and suggested eliminating BCs in immunocompetent patients [5]. Another study showed that of 2210 BCs, only 132 (6%) yielded positive growth [42]. The diagnostic yield of BCs (defined as the yield for true bacteraemia) in our ED was approximately 14% and thus much higher than previously reported [5, 8]. Moreover, when the proportion of ED encounters with BCs taken was increased significantly during the diagnostic ABS period, there was no reduction in the diagnostic yield of BCs, which is an exceptional finding.

In our study, the higher proportion of encounters with BC collection during the diagnostic ABS period led to a higher detection rate of community-onset bacteraemia. The diagnostic ABS intervention bundle also resulted in increased detection of *Pseudomonas aeruginosa* bacteraemia. This was an interesting and important finding, as *Pseudomonas aeruginosa* has evolved into a clinically relevant pathogen of community-onset bacteraemia worldwide [1, 43]. In adults with bacteraemia presenting at the ED, *Pseudomonas aeruginosa* bacteraemia is associated with a higher mortality rate, often due to inappropriate initial empirical treatment [44]. Hence, timely identification of these patients could be critical [45]. In accordance with published evidence [2, 3, 6], our results imply that increasing the number of BCS might lead to better detection of P. aeruginosa bacteraemia.

In conclusion, we believe that our diagnostic stewardship approach resulted in the targeted identification of more patients with bacteraemia by widening indications for the BC diagnostic. The bundled approach of our diagnostic intervention resulted in both a higher proportion of ED encounters with BCs collected and a higher proportion of BC sampling with ≥ 2 BCS obtained. However, we cannot determine to what extent these two factors contributed to the higher detection rate of bacteraemia in the ED. Our data support the hypothesis that the higher detection rate of *Escherichia coli* resulted from a higher general proportion of ED episodes with BCs, whereas *Pseudomonas aeruginosa* detection benefited more from the collection of two BCS. We believe that the presence of a skilled ID team was a reminder of optimised diagnostics and promoted the liberal collection of BCs, which resulted in an improved diagnostic yield. Further, as elevated body temperature is unreliable as an clinical indicator of bacteraemia [41], the recommendation to obtain BCs in the absence of an elevated temperature additionally led to increased awareness.

This study has several limitations. It was a single-centre study based on the retrospective analysis of laboratory data. However, the heterogeneous population of a city-centre-based ED with a large catchment area may allow generalisation to other EDs. Due to the retrospective design of the study, no additional details on the order in which BCs were drawn was available; therefore, it could not be determined in which set growth was established. However, as diagnostic yield is highly dependent on the overall blood volume drawn, the number of sets represents a decent surrogate for volume. No clinical endpoints were available within this study due to its retrospective nature. Therefore, we were unable to report if patient outcomes were actually improved by the timely detection of bacteraemia. However, there is a growing body of evidence that diagnostic stewardship can improve clinical outcomes: rapid diagnostics like expedited processing could also improve outcomes if they were embedded in the ABS by enabling correct interpretation of results and application to treatment decisions [26, 46–49]. Hence, even in the absence of clinical data, we strongly believe that our intervention may result in improved clinical outcomes.

Finally, it should be noted that this intervention was labour intensive as it required specialised staff, and its sustainability would have to be monitored. An automated computer-based clinical decision support system which could flag patients who met the criteria for BCs could be another possible approach, but personal engagement and ID-dedication of the ED physician seemed to be a major factor toward improving the accurate detection of bacteraemia.

## Conclusions

Our findings provided evidence that a diagnostic ABS intervention bundle within the ED could lead to increased detection rates of community-onset bacteraemia. Moreover, identification of relevant pathogens and a detailed analysis of the corresponding susceptibility patterns could lead to improved recommendations for initial empiric treatment. The presence of an ID resident working shifts in the ED and the implementation of a diagnostic ABS intervention bundle resulted in a higher detection rate of true bacteraemia in our study. Earlier detection of relevant pathogens and identification of infectious focus could help provide the basis for earlier targeted antimicrobial therapy and for improved timely management of patients with bacteraemia.

## Supporting information

S1 Table Most relevant microbiological findings. (DOCX)
