# Supplementary material for: Strategies for increasing diagnostic yield of community-onset bacteraemia within the emergency department: A retrospective study
Source: PLoS One. 2019 Sep 12;14(9):e0222545. doi: 10.1371/journal.pone.0222545 (PMC6742407; doi:10.1371/journal.pone.0222545)
Supplement: S1 Table — (DOCX) [file pone.0222545.s001.docx]

|  | Oct 2013 -  Sept 2018  (study period) | Oct 2013 -  Jun 2017  (pre-diagnostic ABS intervention period) | Jul 2017-  Sep 2018  (diagnostic ABS intervention period) |
| --- | --- | --- | --- |
| *Staphylococcus aureus* | 92 | 69 | 23 |
| *Coagulase-negative staphylococci* | 272 | 184 | 88 |
|  |  |  |  |
| *Streptococcus pyogenes* | 16 | 13 | 3 |
| *Streptococcus agalactiae* | 19 | 10 | 9 |
| *Streptococcus dysgalactiae* | 8 | 6 | 2 |
| *Streptococcus pneumoniae* | 34 | 24 | 10 |
| *Streptococcus anginosus* | 13 | 11 | 2 |
| *Streptococcus intermedius* | 6 | 5 | 1 |
| *Streptococcus constellatus* | 4 | 2 | 2 |
| *Enterococcus faecalis* | 18 | 13 | 5 |
| *Enterococcus faecium* | 12 | 8 | 4 |
| *Enterococcus casseliflavus* | 3 | 2 | 1 |
|  |  |  |  |
| *Escherichia coli* | 316 | 210 | 106 |
| *Klebsiella pneumoniae* | 48 | 36 | 12 |
| *Klebsiella oxytoca* | 16 | 13 | 3 |
| *Klebsiella variicola* | 2 | 0 | 2 |
| *Morganella morganii* | 5 | 4 | 1 |
| *Enterobacter cloacae* | 16 | 10 | 6 |
| *Enterobacter aerogenes* | 4 | 3 | 1 |
| *Enterobacter amnigenus* | 1 | 1 | 0 |
| *Proteus mirabilis* | 9 | 6 | 3 |
| *Proteus vulgaris* | 1 | 1 | 0 |
| *Serratia marcescens* | 6 | 5 | 1 |
| *Raoultella (Klebsiella) ornithinolytica* | 5 | 5 | 0 |
| *Salmonella enteritidis* | 1 | 0 | 1 |
| *Salmonella typhimurium* | 1 | 1 | 0 |
| *Citrobacter freundii* | 3 | 3 | 0 |
| *Citrobacter koseri / diversus* | 4 | 3 | 1 |
| *Hafnia alvei* | 1 | 1 | 0 |
|  |  |  |  |
| *Haemophilus influenzae* | 5 | 4 | 1 |
| *Pseudomonas aeruginosa* | 32 | 18 | 14 |
| *Bacteroides spp.* | 12 | 7 | 5 |

**Supplementary Table 1. Most relevant microbiological findings**
